# Review on manganese carbon interactions in manganese oxide-modified biochar for environmental remediation

**DOI:** 10.1016/j.isci.2025.113991

**Published:** 2025-11-13

**Authors:** Anyu Li, Song Lei, Shujia Chen, Xiaotong Zhou, Zhihua Wu, Feng Jiang, Peng Zheng, Lening Hu, Hua Deng

**Affiliations:** 1 Guangxi Key Laboratory of Environmental Processes and Remediation in Ecologically Fragile Regions, Guangxi Normal University, Guilin 541004, China; 2 University Engineering Research Center of Green Remediation and Low Carbon Development for Lijiang River Basin, Guangxi Normal University, Guilin 541004, China; 3 Key Laboratory of Ecology of Rare and Endangered Species and Environmental Protection, Guangxi Normal University, Guilin 541004, China; 4 College of Environment and Resources, Guangxi Normal University, Guilin 541004, China; 5 Key Laboratory of Environmental Remediation and Ecological Health, Ministry of Industry and Information Technology, School of Environmental and Biological Engineering, Nanjing University of Science and Technology, Nanjing, China

**Keywords:** Environmental science, Pollution, Materials science

## Abstract

Manganese oxide-modified biochar (MBC) has attracted much attention as a multifunctional environmental remediation material. This work systematically examines the remediation mechanisms of MBC and its applications in environmental pollution control, with a focus on its potential for adsorbing and immobilizing soil and water pollutants. We provide a detailed analysis of the relevant interfacial reaction processes, including charge interactions, surface coordination, redox reactions, and ion exchange, emphasizing their roles in pollutant remediation and degradation. In terms of environmental applications, this review summarizes the research progress on the utilization of MBC in the treatment of wastewater and contaminated soils, with a particular focus on its effectiveness in removing these pollutants. This work establishes a theoretical basis for the utilization of MBC as a highly efficient and sustainable adsorption material for environmental pollution control and provides a scientific reference for further research on its interfacial mechanisms and practical application.

## Graphical abstract

**Figure:**
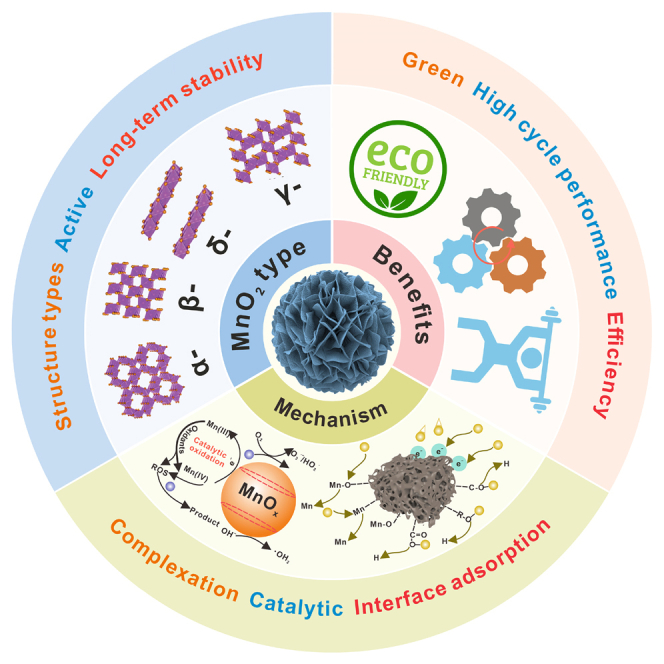

## Introduction

The expansion of industrial activities related to mining, metallurgy, and chemical engineering, as well as agricultural growth, which is frequently linked to the uncontrolled application of agricultural chemicals, have led to the release of significant quantities of heavy metals and organic pollutants into the environment.^1^^,^^2^^,^^3^ These contaminants diffuse through various media, including air, water, and soil, thereby threatening both ecosystems and human health. Certain toxic metals, including arsenic (As), cadmium (Cd), and antimony (Sb), are characterized by their bioaccumulation and persistence, which enables their progressive magnification through the food chain, ultimately causing neurological damage, organ damage, cancer, and other problems in humans.^4^^,^^5^ Organic pollutants, including polycyclic aromatic hydrocarbons (PAHs), present risks comparable to those of heavy metals and can be readily absorbed through human skin, the respiratory tract, and the digestive system.^6^^,^^7^^,^^8^ Currently, the primary techniques for remediating soil and water pollution include bioremediation, chemical complexation, electrosorption, and membrane-based treatments.^9^^,^^10^^,^^11^ Nevertheless, these methods are frequently hindered by challenges such as high operational costs, complex procedures, low efficiency, and limited recyclability, which impede their ability to meet large-scale remediation requirements. Among various remediation techniques, the adsorption method offers significant advantages, including high efficiency, low cost, and ease of operation, while minimizing the risk of environmental pollution. Consequently, it is increasingly recognized as a key technology for addressing environmental contamination.^12^^,^^13^ The principle of adsorption involves removing heavy metals from environmental media on the basis of physical or chemical interactions with adsorbents, including graphene, natural minerals, and various industrial and agricultural wastes.^14^^,^^15^^,^^16^ Among the various types of adsorbents, biochar, a low-cost, carbon-rich functional material produced from agricultural waste, has a porous structure, abundant surface functional groups, and tunable surface properties, making it a potential remediation material for environmental pollution. However, the wide range of biomass sources results in significant variations in the adsorption performance of biochar produced from different feedstocks. For example, a rice husk-derived biochar presented a relatively low adsorption performance of 9.9 mg g^−1^ for Cd, primarily due to its strong hydrophobicity, limited functional group activity, and lack of active sites^17^; a crayfish shell-derived biochar, recognized as a heavy metal deactivator, was reported to have an adsorption capacity of 1166.4 mg g^−1^ for Pb.^18^ Furthermore, biochar derived from sources such as iron sludge and feces contains heavy metals and organic pollutants, and if it is used without further treatment, environmental contamination can be exacerbated. To address the limitations of unmodified biochar, physical and chemical modification techniques, including ball milling, microwave treatment, acid/alkali activation, and doping, are employed to enhance its performance. For instance, microwave activation enhances the microporous/mesoporous structure of biochar, whereas alkaline activation generates multiple functional groups that increase the pH, facilitate heavy metal precipitation, and improve the remediation potential and effectiveness in acidic environments.^14^ From a cost perspective, physical activation is associated with high energy consumption, prolonged activation cycles, and limited environmental benefits; conversely, chemical activation presents challenges such as a high risk of secondary pollution, complex procedures, and poor selectivity in adsorption. Unlike other modification methods, the incorporation of metal oxides such as Fe, Mn, and Mg not only increases the specific surface area but also significantly enhances their pore structure, thereby improving their performance in treating and immobilizing environmental pollutants. Moreover, catalytic metal oxides can be incorporated to enhance the adsorption of anionic heavy metals and facilitate the degradation of organic matter via redox reactions, thereby converting heavy metal ions into more stable, less toxic forms while organic matter is degraded into smaller molecular compounds. When utilizing metal oxides, careful consideration must be given to whether the introduced metals pose environmental risks, particularly during large-scale production, and to the effective management and treatment of the resulting chemical waste.

Manganese oxides (MnO_x_) occur in various forms in natural environments, including MnO_2_ and Mn_2_O_3_; these oxides are widely distributed in soil, water, and sediment with high geochemical abundance, particularly in environments with significant fluctuations in redox conditions.^19^^,^^20^^,^^21^ Different MnO_x_ types exhibit diverse structures, varying oxidizing properties, and specific surface areas and are key to the sequestration and elimination of anionic chromium (Cr) and arsenic (As) through redox reactions, contributing significantly to both chemical and biological processes in the environment.^19^ In the remediation of heavy metal pollution, the high surface activity and porous structure of MnO_x_ offer numerous sites, facilitating the physical adsorption of heavy metal ions. Additionally, MnO_x_ surfaces are decorated with abundant hydroxyl (-OH) and manganese−oxygen (Mn−O) bonds, which form stable inner-sphere complexes with heavy metal ions, further increasing the adsorption efficiency. The negative charge on the surface of MnO_x_ facilitates the adsorption of cations via electrostatic attraction, further stabilizing these heavy metals through ion exchange or surface complexation. Moreover, certain MnO_x_ materials exhibit redox properties, enabling the formation of hydrogen or covalent bonds with anions via surface hydroxyl groups or the oxidation of heavy metal anions to higher valence states through redox reactions, leading to their precipitation. For instance, β-MnO_2_ can oxidize As(III) to As(V), a form that is more prone to forming insoluble precipitates in the environment, thereby enabling its effective removal.^22^ Therefore, MnO_x_ has distinctive adsorption properties and offers several advantages, including multifunctional mechanisms including complexation, redox reactions, and precipitation, making it highly effective in immobilizing environmental pollutants. The multifunctionality of MnO_x_ is demonstrated by its adaptability to varying environmental conditions, as it maintains high stability under acidic, neutral, and alkaline conditions, which enhances its broad applicability in environmental remediation. While the high surface activity of MnO_x_ can lead to aggregation, it also facilitates the adhesion of MnO_x_ to the surface of biochar through specific interactions, thereby enhancing the adsorption capacity and environmental adaptability. For example, Zhang et al.^23^ utilized KMnO_4_ to react with the carbon in biochar, directly producing δ-MnO_2_, which efficiently immobilizes heavy metals in soil, transforming them into stable crystalline manganese and iron oxides.

However, few reviews have focused on MBC; most of the existing reviews address its preparation, characterization, and applications. For example, the review conducted by Zhu et al.^19^ provided a comprehensive summary of the synthesis, preparation methods, and applications of MBC and outlined the influence of different manganese oxide structures. Nevertheless, it offered a limited discussion of the mechanistic pathways governing the formation of distinct manganese oxide polymorphs, leaving a critical gap in the current understanding. Shaheen et al.^24^ further emphasized the preparation and characterization of MBC, highlighting that its adsorption potential is governed by feedstock characteristics, pyrolysis temperature, and modification ratio, as these parameters induce significant alterations in morphology, surface functional groups, and elemental composition. Notably, there is a lack of comprehensive reviews on biochar modification via the loading of manganese oxides with various structural forms, particularly regarding how the form of manganese oxide used significantly affects biochar performance. Numerous studies have investigated the adsorption mechanisms of MBC for heavy metals and organic pollutants, which include complexation, redox reactions, and precipitation; however, the relative contributions of these mechanisms to the adsorption of different pollutants remain unclear, so further reviews are necessary to guide future research. Moreover, biomass type significantly influences the loading process; however, comprehensive reviews focusing on the regeneration, reuse, and economic sustainability of manganese-modified biochar remain limited. The review highlights the interfacial mechanisms and environmental applications of MBC, emphasizing their multifunctional roles in immobilizing heavy metals and organic pollutants. We further discuss performance evaluation and stability assessment methods, providing insights into the design of efficient and sustainable MBC-based remediation materials.

## Physicochemical properties of manganese oxides

**Figure 1:**
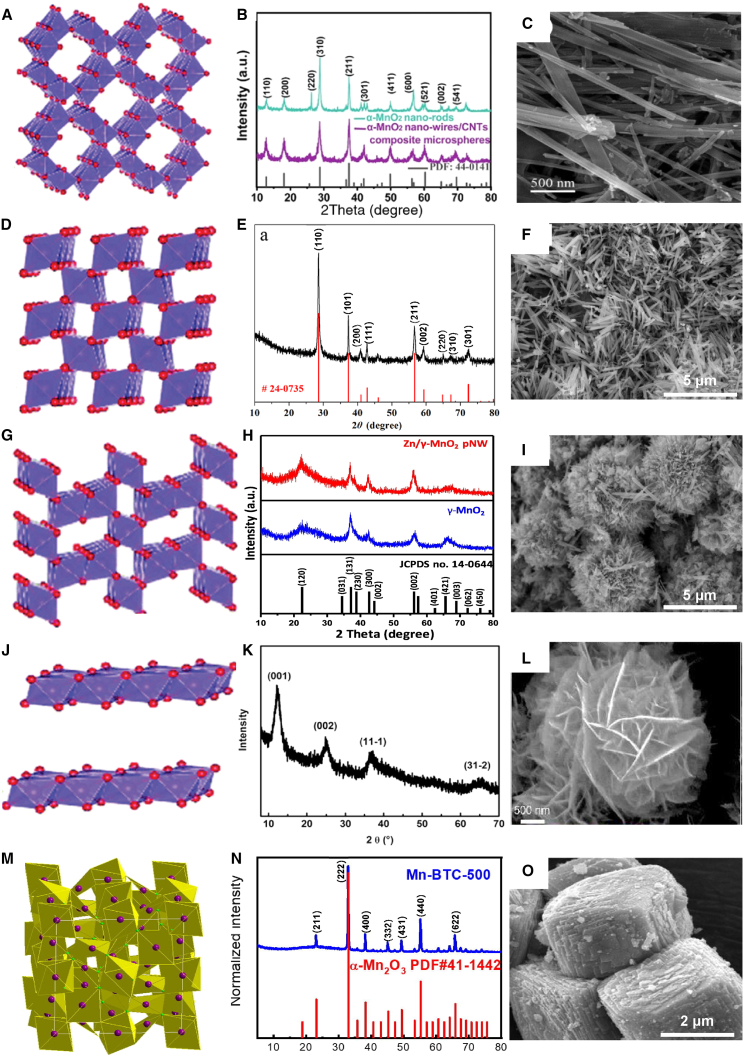
Structural characteristics of different manganese oxides Crystalline structures, XRD patterns, and SEM images of (A–C) α-MnO_2_.^34^^,^^35^ (D–F) β-MnO_2_.^35^^,^^36^^,^^37^ (G–I) γ-MnO_2_.^35^^,^^36^^,^^38^ (J–L) δ-MnO_2_.^35^^,^^39^^,^^40^ (M–O) Mn_2_O_3_.^41^^,^^42^ Copyright Elsevier, OAE, and American Chemical Society.

Mn, ranked the tenth most abundant element in the crust of Earth and the second most abundant transition metal after Fe, has an abundance of 0.085%. The Mn content in soil generally ranges from 500 to 1000 mg kg^−1^, approximately 10% of the iron content.^25^ The most common valence states of Mn are +2, +3, and +4, which impart redox properties to Mn, enabling it to exhibit geochemical behavior in soil environments.^26^ Under reducing conditions, Mn primarily exists as highly soluble Mn^2+^, whereas in oxidizing environments, it forms insoluble oxides such as MnO_2_, Mn_2_O_3_, and Mn_3_O_4_; additionally, Mn can combine with carbonates, silicates, and organic matter to form various compounds, leading to significant variations in the physicochemical properties of manganese oxides across different valence states. Figure 1 shows the types of manganese oxides commonly used for loading onto modified biochar, which include α-, β-, λ-, and δ-MnO_2_. In particular, α-MnO_2_ features a 2 × 2 tunnel structure formed by interconnected MnO_6_ octahedra, which can accommodate larger cations such as Ba^2+^ and K^+^, imparting high stability and a moderate oxidation state; this material exhibits characteristic (110), (200), (220), and (310) crystal planes and a typical granular or needle-like surface morphology^27^ (Figures 1A–1C). Zhang et al.^28^ reported that an α-MnO_2_-modified biochar composite effectively removed As through both oxidation and adsorption; not only did the biochar have an excellent specific surface area, but the results confirmed that Mn-OH was the primary functional group responsible for the adsorption and immobilization of As. Furthermore, studies have shown that during As adsorption by α-MnO_2_-modified biochar, stable complexes form on the (100) and (110) crystal planes, with the stability of the former being greater than that of the latter.^29^ Compared with other forms, β-MnO_2_ has a more stable structure, with a denser surface and smaller grain size, and typically displays block, needle, or flake morphologies. Although β-MnO_2_ has a smaller porosity and a more uniform surface with lower reactivity than other forms of manganese oxide, it is utilized primarily in battery cathode materials, catalysts, and water treatment agents, with limited application in biochar modification (Figures 1D–1F). γ-MnO_2_ has an irregular and disordered structure, typically forming nanoscale particle aggregates, and is often a phase mixture of β-MnO_2_ and its variants with a certain proportion of Mn^3+^, resulting in a mixed valence state. γ-MnO_2_ has high redox activity and strong oxidative properties, particularly in electrochemical reactions (Figures 1G–1I). Manganese(III) oxide (Mn_2_O_3_), consisting of two manganese atoms and three oxygen atoms with manganese in the +3 oxidation state, is an intermediate oxide that can be further oxidized to MnO_2_ or reduced to MnO or elemental manganese and typically has a cubic or hexagonal crystal structure (Figures 1M–O); therefore, it is highly active in redox reactions. He et al.^30^ employed ball milling to integrate Mn_2_O_3_ with biochar for the removal of sulfamethoxazole; a removal efficiency exceeded 95% within a short duration, and the process was unaffected by coexisting ions. Unlike the manganese oxides discussed above, δ-MnO_2_ features a layered structure composed of MnO_6_ octahedral sheets. Weak van der Waals forces or hydrogen bonds exist between the layers, allowing the incorporation of water molecules and cations (K^+^ and Na^+^), which endows the material with a high ion exchange capacity. Conventional δ-MnO_2_, characterized by poor crystallinity, small crystal particles, a large number of defects and irregular shapes, features highly active chemical sites at grain boundaries, dislocations, and vacancy defects, resulting in superior adsorption performance compared with that of other types of manganese oxides^31^^,^^32^ (Figures 1J–L). In summary, δ-MnO_2_ exhibits excellent ion exchange capacity and redox activity, particularly in aquatic environments, where it demonstrates strong adsorption capabilities and effective removal of heavy metals.^33^

## Preparation of manganese oxide-modified biochar

**Figure 2:**
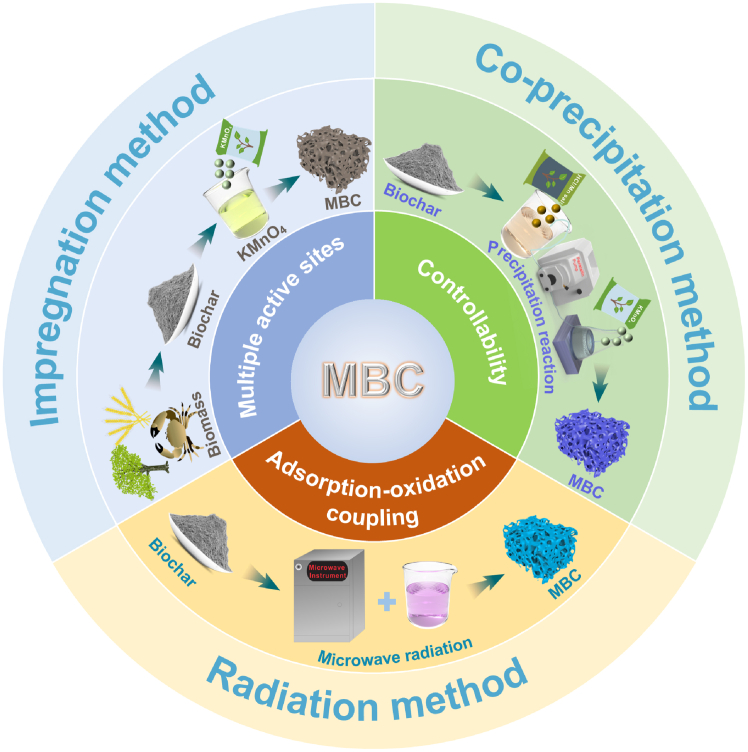
Schematic diagram of the procedure for fabricating MBC MBC represents the manganese oxide-modified biochar obtained by different methods.

Various modification techniques yield different loadings of manganese oxides, resulting in significant variations in the mechanisms and properties of the prepared materials. The commonly employed methods include solution impregnation, coprecipitation, pyrolysis, chemical deposition, and ultrasound-assisted techniques. Figure 2 and Table 1 illustrate the effects of material type, carbonization temperature, solid-liquid ratio, and synthesis principle on the types of manganese oxides. Notably, variations in the biochar modification conditions can significantly affect the loading of manganese oxides. Overall, MBC prepared by different synthesis methods exhibits multiple active sites, high structural controllability, and combined adsorption-oxidation properties.

**Table 1:** Synthesis methods of manganese oxide modified-biochar using different techniques

Biomass	Pyrolysis temperature	Solid-liquid ratio	Synthetic process	Principle	Type	Reference
Maize straw	450 °C	1:12	KMnO_4_ and MnSO_4_ are formed through redox reactions in acidic environments ${\text{MnO}}_{4}^{-}+8H^{+}+5e^{-}\to Mn^{2+}+4H_{2}O$ $Mn^{2+}+2H_{2}O\to \alpha -\text{Mn}O_{2}+4H^{+}+2e^{-}$	Coprecipitation	α-MnO_2_	Zhang et al.^28^
Hickory chips	600 °C	1:5	Redox reaction between KMnO_4_ and carbon in biomass under radiation conditions $4{\text{MnO}}_{4}^{-}+3C+H_{2}O\to 4\delta -{\text{MnO}}_{2}+{\text{CO}}_{3}^{2-}+2{\text{HCO}}_{3}^{-}$	Radiation	δ-MnO_2_	Wang et al.^43^
Ficus macrocarpa aerial root	800 °C	1:100	KMnO_4_ and Mn(NO_3_)_2_ undergo redox reactions in an acidic environment ${\text{MnO}}_{4}^{-}+3{\text{Mn}}^{2+}+4H^{+}\to \delta -{\text{MnO}}_{2}+2H_{2}O+3{\text{Mn}}^{3+}$	Coprecipitation	δ-MnO_2_	Liu et al.^33^
Grapefruit peel	800 °C	1:30	KMnO_4_ and MnSO_4_ undergo redox reactions in an acidic environment $\begin{array}{l} {\text{MnO}}_{4}^{-}+8H^{+}+5e^{-}\to {\text{Mn}}^{2+}+4H_{2}O\\ {\text{Mn}}^{2+}+2H_{2}O\to \alpha -{\text{MnO}}_{2}+4H^{+}+2e^{-} \end{array}$	Coprecipitation	α-MnO_2_	Zhou et al.^44^
Peanut shell	400 °C	–	NaClO oxidizes MnSO_4_ in an alkaline environment 2Mn^2+^+2ClO^−^+4OH^−^→2γ-MnO_2_+2H_2_O + Cl_2_↑	Coprecipitation	γ-MnO_2_	Wan et al.^45^
Rice husk and wood chip	400 °C	1:10	KMnO_4_ undergoes redox reaction with HCl $2{\text{MnO}}_{4}^{-}+10{\text{Cl}}^{-}+16H^{+}\to 2\delta -{\text{MnO}}_{2}+8H_{2}O+5{\text{Cl}}_{2}↑$	Coprecipitation	δ-MnO_2_	Lee and Shin^46^
Loblolly pine	600 °C	7:50	Mn^2+^ reacts with O_2_ in a high temperature environment 6Mn^2+^+O_2_→2Mn_3_O_4_	Pyrolysis	Mn_3_O_4_	Wang et al.^47^
		1:10	KMnO_4_ undergoes redox reaction with HCl $2{\text{MnO}}_{4}^{-}+10{\text{Cl}}^{-}+16H^{+}\to 2\delta -{\text{MnO}}_{2}+8H_{2}O+5{\text{Cl}}_{2}↑$	Coprecipitation	δ-MnO_2_	
Corn straw	600 °C	–	KMnO_4_ decomposes into MnO_2_ at high temperature 6KMnO_4_→2Mn_3_O_4_+2K_2_O+5O_2_↑	Pyrolysis	γ-MnO_2_	Yu et al.^48^
Water hyacinth	450 °C	1:25	KMnO_4_ is reduced by H_2_O_2_ in an acidic environment $2{\text{MnO}}_{4}^{-}+5H_{2}O_{2}+6H^{+}\to 2{\text{MnO}}_{2}+8H_{2}O+5O_{2}↑$	Coprecipitation	δ-MnO_2_	Zhang et al.^49^
Rice husk	600 °C	1:50	KMnO_4_ decomposes in high temperature environment 6KMnO_4_→2Mn_3_O_4_+2K_2_O+5O_2_↑	Pyrolysis	Mn_3_O_4_	Sun et al.^50^
Sawdust and swine manure	400 °C	1:10	KMnO_4_ reacts with carbon in biochar and oxidizes Mn^2+^ $4{\text{MnO}}_{4}^{-}+3C+H_{2}O\to 4\delta -{\text{MnO}}_{2}+{\text{CO}}_{3}^{2-}+2{\text{HCO}}_{3}^{-}$ $2{\text{Mn}}^{2+}+2{\text{MnO}}_{4}^{-}+2H_{2}O\to 5\delta -{\text{MnO}}_{2}+4H^{+}$	Coprecipitation	δ-MnO_2_	Liang et al.^51^
Wheat straw	500 °C	1:10	Reaction of KMnO_4_ with carbon in biochar $2{\text{MnO}}_{4}^{-}+4C\to 2\delta -{\text{MnO}}_{2}+{\text{CO}}_{3}^{2-}+3{\text{CO}}_{2}↑$	Pyrolysis	δ-MnO_2_	Xu et al.^52^
Rice husk	500 °C	1:10	KMnO_4_ oxidizes Mn^2+^ $2{\text{Mn}}^{2+}+2{\text{MnO}}_{4}^{-}+2H_{2}O\to 5\delta -{\text{MnO}}_{2}+4H^{+}$	Coprecipitation	δ-MnO_2_	Wu et al.^53^
Cotton stem	600 °C	1:1	KMnO_4_ decomposes in high temperature environment 6KMnO_4_→2Mn_3_O_4_+2K_2_O+5O_2_↑	Pyrolysis	Mn_3_O_4_	Chang and Li^54^
Raw spinach	600 °C	–	Reaction of KMnO_4_ with carbon in biochar and the presence of high temperature oxidation side reaction $2{\text{MnO}}_{4}^{-}+5C\to 2\delta -{\text{MnO}}_{2}+{\text{CO}}_{3}^{2-}+3{\text{CO}}_{2}↑$ 3MnO_2_→Mn_3_O_4_+O_2_↑	Pyrolysis	Mn_3_O_4_ and α-MnO_2_	Chen et al.^55^
Rape straw	600 °C	1:10	Reaction of KMnO_4_ with carbon in biochar and the presence of high temperature oxidation side reaction $2{\text{MnO}}_{4}^{-}+5C\to 2\delta -{\text{MnO}}_{2}+{\text{CO}}_{3}^{2-}+3{\text{CO}}_{2}↑$	Coprecipitation	δ-MnO_2_	Li et al.^56^
Chestnut shell	700 °C	–	KMnO_4_ undergoes redox reaction with HCl $2{\text{MnO}}_{4}^{-}+10{\text{Cl}}^{-}+16H^{+}\to 2{\text{MnO}}_{2}+8H_{2}O+5{\text{Cl}}_{2}↑$	Coprecipitation	δ-MnO_2_ and γ-MnO_2_	Dai et al.^57^
Wheat straw	400°C and 600°C	–	KMnO_4_ and MnSO_4_ undergo redox $2{\text{MnO}}_{4}^{-}+3{\text{Mn}}^{2+}\to 5{\text{MnO}}^{2}+4H^{+}$	Coprecipitation	δ-MnO_2_	Huang et al.^58^
Pharmaceutical sludge	600 °C	1:5	Redox reaction between KMnO_4_ and carbon in biomass under radiation conditions $4{\text{MnO}}_{4}^{-}+3C+2H_{2}O\to {\text{Mn}}_{3}O_{4}+4{\text{OH}}^{-}+3{\text{CO}}_{2}↑$	Radiation	Mn_3_O_4_	Cui et al.^59^
Bamboo	500 °C	1:25	Mn^2+^ is oxidized to high-valent manganese in a high temperature environment 6Mn^2+^+4O_2_→2Mn_3_O_4_	Pyrolysis	Mn_3_O_4_	Su et al.^60^
Rice husk	500 °C	1:20	KMnO_4_ decomposes in high temperature environment 6KMnO_4_→2Mn_3_O_4_+2K_2_O+5O_2_↑	Pyrolysis	Mn_3_O_4_	Shao et al.^61^
Rape straw	500 °C	1:100	Reaction of KMnO_4_ with carbon in biochar $4{\text{MnO}}_{4}^{-}+3C+H_{2}O\to 4\delta -{\text{MnO}}_{2}+{\text{CO}}_{3}^{2-}+2{\text{HCO}}_{3}^{-}$	Coprecipitation	δ-MnO_2_	Gao et al.^62^

Solution impregnation is a widely used technique for modifying biochar with manganese oxides. In this method, the biochar precursor is immersed in a solution containing manganese salts (such as MnSO_4_, MnCl_2_, and KMnO_4_), and the salts adhere to the surface or pore structure of the biochar via physical adsorption or chemical reactions.^63^^,^^64^^,^^65^ The desired manganese oxide loading can be achieved by controlling factors such as impregnation time and manganese salt content, while the type, purity, and crystal structure of the manganese oxide can be further refined through pyrolysis or chemical reduction after impregnation. Zhang et al.^23^ immersed biomass in KMnO_4_ solution under heating to facilitate the reaction between carbon and KMnO_4_ to generate δ-MnO_2_. The desired manganese oxide was synthesized without pyrolysis, while the weakly crystalline layered structure exposed additional defect sites. The structure of manganese oxide is changed by controlling the pH of the impregnation solution and the reaction temperature, which facilitates the transformation of δ-MnO_2_ into γ-MnO_2_. Moreover, the reaction equation relating carbon and KMnO_4_ can be used to calculate and adjust the solid‒liquid ratio, thereby maximizing cost efficiency.^66^ In contrast to KMnO_4_ immersion, the use of MnSO_4_ and MnCl_2_ for impregnation typically necessitates pyrolysis to convert the loaded Mn^2+^ into manganese oxides. This process may yield multiple types of manganese oxides, which complicates subsequent analysis of the underlying mechanisms. Furthermore, the pyrolysis process typically oxidizes Mn^2+^ to Mn_3_O_4_, which possesses good crystallinity; however, its adsorption performance is generally inferior to that of weakly crystalline manganese oxides.^60^ In summary, the solution impregnation method simplifies the process, offering advantages such as ease of operation, low cost, and strong controllability, making it ideal for large-scale production and processing. Furthermore, optimizing the impregnation conditions, particularly the hydrothermal environment, further increases the efficiency and stability of the loading process.^67^^,^^68^ However, while the solution impregnation method can partially control the morphology and loading amount of manganese oxides, precisely controlling their size and morphology remains challenging, and the resulting biochar may contain multiple forms of manganese oxides.

The coprecipitation method involves introducing a reducing or oxidizing agent into solution to induce manganese oxide precipitation on the biochar surface while adjusting the reaction time, temperature, and pH to control the morphology and size of the product, thereby regulating the surface structure and function of the biochar.^69^^,^^70^ The relevant reaction equations can be referenced to encapsulate highly reactive and versatile manganese oxides on the biochar surface, which enables good control of the process. For example, Liu et al.^33^ utilized KMnO_4_ and Mn(NO_3_)_2_ in an acidic environment to facilitate a redox reaction, leading to the production of weakly crystalline δ-MnO_2_. The resulting manganese oxide exhibited an enhanced pore structure and carbon skeleton and thus more exposed active sites. Another method involves directly reacting HCl with a suspension containing biochar and KMnO_4_ to generate δ-MnO_2_. However, the process must be performed slowly to prevent excessive byproduct formation or overly intense reactions, and multiple washings are required to remove the byproducts and residues, which increases both the preparation cost and process complexity.^47^ In contrast to solution impregnation, coprecipitation simultaneously forms metal oxides and carriers during the reaction, ensuring the uniform distribution of manganese oxides both on the surface and inside the biochar. This feature is a key factor in enhancing adsorption performance and reactivity. Moreover, the manganese oxide loading in biochar can be accurately controlled based on the solution concentration and conditions, whereas the solution impregnation method is less effective in this regard. This approach also effectively prevents the degradation of manganese oxide during pyrolysis, which would otherwise result in changes in its crystal structure. Although coprecipitation offers certain advantages, the complex reaction process and the need for precise control of the reaction conditions necessitate further optimization for practical application.

Radiation-assisted methods can also modify and load manganese oxides. These methods primarily employ radiation sources such as gamma rays, electron beams, or ultraviolet light to promote the surface modification of biochar and the incorporation of functional materials.^59^ During irradiation, free radicals produced by high-energy radiation can initiate chemical reactions on the biochar surface to promote the combination of the biochar with the modifier. This technique also enables material modification at lower temperatures and allows for precise control of the radiation dose and exposure time, thereby enhancing the material loading efficiency and uniformity. For instance, free radicals generated by gamma rays can rapidly react with MnSO_4_ or KMnO_4_ precursors to promote the formation and deposition of well-dispersed manganese oxides. Additionally, radiation can alter the chemical compositions on the biochar surface, facilitating the formation of stronger chemical bonds with the loaded manganese oxides or other functional materials and thereby enhancing the stability and longevity of the optimized biochar. Radiation-assisted treatments show promise in the preparation of environmental materials, particularly in heavy metal adsorption and organic matter degradation. Such methods also promote the production of a more complex pore structure on the surface of biochar, increasing the complexation and catalytic performance. In terms of environmental benefits, while the equipment cost for radiation-assisted technology is relatively high, this approach enables rapid material modification, reduces energy consumption, and minimizes chemical waste production. However, before large-scale applications can be realized, issues such as the cost and safety of radiation equipment, reaction controllability and uniformity, and process standardization must be addressed. In practice, the choice of manganese oxide modification method should be guided by the intended application, cost, and desired structural properties. Solution impregnation is recommended when simplicity, scalability, and cost efficiency are prioritized, especially for large-scale production. Coprecipitation is more suitable for applications requiring uniform dispersion and greater control over the crystalline phase of manganese oxides, although precise reaction control is essential. Radiation-assisted techniques offer unique advantages in producing well-dispersed and stable manganese oxides with enhanced catalytic performance, but the high equipment cost currently limits their practical application. Therefore, we should select modification methods by balancing the preparation complexity, structural uniformity, cost, and target pollutants to be remediated.

## Effect and selection of biomass type

Biomass is an organic material derived from plants, animals, and their byproducts and are formed primarily via photosynthesis. It encompasses organic matter produced and decomposed through natural processes and is a key factor influencing the physicochemical properties of biochar.^71^^,^^72^^,^^73^ Biomass typically includes agricultural waste and organic waste. It is renewable and can be converted into energy or organic materials through processes such as combustion and fermentation. The properties of various biomass types exhibit considerable variation; for example, agricultural biomass, which is commonly used for fuel, fertilizer, or biomass energy production, exhibits high cellulose and hemicellulose contents. However, improper handling can lead to resource waste and environmental pollution.^74^ Livestock and poultry manure, such as cow, pig, and chicken manure, is rich in organic matter, nitrogen, and phosphorus, making it suitable for the preparation of biochar-based fertilizers; however, it also readily generates foul-smelling gases, which can lead to environmental pollution.^75^ Biochar derived from organic waste contains numerous hydrophilic groups and is rich in nutrients, but it exhibits low environmental stability, a high risk of nutrient loss, and significant amounts of volatile organic matter residues on its surface.^76^ The metal and alkaline oxides (such as CaO, Na_2_O, Fe_2_O_3_, and MgO) in biochar produced from industrial waste can enhance the adsorption performance. However, pyrolysis may lead to the accumulation of significant amounts of pollutants, which can be released as the environmental conditions change, and the resulting biochar is also prone to degradation by microorganisms, reducing its long-term effectiveness. Biomass forms biochar and other byproducts with varying physicochemical properties depending on the pyrolysis temperature. For instance, during low-temperature pyrolysis of agricultural waste (200°C–400°C), cellulose and hemicellulose are not completely decomposed, and some volatile organic matter remains in the biochar. Although agricultural waste-derived biochar contains large amounts of carboxyl (-COOH) and hydroxyl (-OH) groups, its adsorption activity remains relatively low, hindering the complexation of heavy metals.^23^^,^^77^ Although biochar produced in low-temperature environments exhibits good hydrophilicity, its pore structure is underdeveloped, its specific surface area is low, and its structural stability is poor, all of which affect the loading efficiency of manganese oxides. In contrast, during high-temperature pyrolysis (400°C–700°C), the cellulose and hemicellulose are more thoroughly decomposed, and the biochar exhibits greater structural stability. This process enhances the development of micro- and mesoporous structures in biochar, thereby increasing its specific surface area and overall pore volume while also allowing the biochar to remain stable in the environment over time. High-temperature treatment is particularly suitable for loading manganese oxides, as it helps preserve the hydrophobicity of the biochar; however, the temperature must be carefully controlled to avoid excessive costs. Therefore, selecting the appropriate raw biomass material is crucial for improving the remediation effect, reducing costs, and ensuring long-term stability.

When biomass is selected for the preparation of the biochar used in environmental remediation, the following principles can be considered: (i) agricultural residues are suitable for applications requiring abundant surface functional groups but should be combined with appropriate modification strategies to overcome their structural limitations; (ii) livestock and poultry manure is advantageous for producing nutrient-rich biochar-based fertilizers; however, additional pretreatment is needed to reduce odor emissions and eliminate potential pathogens; (iii) organic waste can yield biochar with high nutrient content, but stabilization treatment is necessary to mitigate nutrient leaching and residual volatile organic compounds; (iv) industrial waste biomass, due to its inherent metal and alkaline oxide content, can enhance adsorption, but strict control of pyrolysis and post-treatment processes is essential to prevent the release of hazardous pollutants; and (v) pyrolysis temperature should be optimized according to the target remediation purpose, with low-temperature products favoring functional group retention and high-temperature products offering greater structural stability and surface area. Therefore, the rational selection and pretreatment of biomass feedstocks, combined with controlled pyrolysis, are critical for tailoring the properties of biochar for the efficient and sustainable remediation of contaminated environments.

## Characteristics of manganese oxide-modified biochar

### Properties of manganese oxide-modified biochar

Manganese oxides are primarily embedded in pores on the biochar surface or react with surface elements such as carbon, and different types of manganese oxides lead to distinct morphologies. As introduced in the second part, the surface of α-MnO_2_-modified biochar typically has a needle, rod, or nanofiber morphology. In contrast, Mn_3_O_4_- and β-MnO_2_-modified biochar exhibits nanorod or particle shapes with a compact structure, whereas γ-MnO_2_-modified biochar mostly forms flakes or irregular clusters. In contrast, δ-MnO_2_-modified biochar typically exhibits a flake or layered structure, resembling a flower-like morphology or stacked flakes. Moreover, the type of manganese oxide can be further identified by analyzing the XRD peak positions of standard substances corresponding to the characteristic crystal planes. Generally, after modification, α-MnO_2_ has characteristic peaks corresponding to the (310), (211) and (521) crystal planes at approximately 29°, 37° and 60°, while β-MnO_2_ and γ-MnO_2_ have characteristic peaks corresponding to the (110)/(120), (101)/(131) and (211)/(002) crystal planes at approximately 28°/22°, 37°/37° and 39°/39°, respectively.^78^ Moreover, δ-MnO_2_-modified biochar with a weak crystalline structure has many exposed active sites, with characteristic peaks attributed to the (001), (002), (111), and (311) crystal planes typically appearing at 11°, 24°, 37°, and 66°, respectively.^23^ To better evaluate the performance of MBC, its adsorption efficiency is determined by not only the crystalline type of manganese oxides but also parameters such as the loading amount, pollutant type, concentration, and treatment duration. For example, layered δ-MnO_2_, with abundant defects and high surface activity, is highly efficient for the immobilization of heavy metals such as Pb, Cd, and Cr for short-to-medium treatment times. From a performance perspective, increasing the manganese oxide content generally increases the density of active sites and pollutant removal efficiency; however, excessive loading may block biochar pores, reduce the specific surface area, and hinder adsorption. In general, MBC exhibits a higher adsorption capacity when the loading ratio is maintained at 5–20 wt %, as excessive loading tends to cause aggregation and reduce the number of accessible active sites. Similarly, higher pollutant concentrations can accelerate adsorption kinetics initially but may saturate active sites, requiring the optimization of the content and contact time to maintain efficiency. When comparing the adsorption performance of MBC modified with different manganese oxides, the aforementioned conditions should be controlled, and all manganese oxides types should be evaluated for the same loading amount.

### Changes in physicochemical properties after modification

Manganese oxide loading influences the elemental composition of biochar. For example, Shaheen et al.^24^ reported that the contents of C, H, O, and N in unmodified biochar were 51.8%, 2.6%, 14.6%, and 1.5%, respectively, with no detectable Mn. After modification, an increase in Mn loading led to a pronounced decrease in C content, with the Mn content reaching a maximum of 27.2%, while the pore volume of the biochar was enhanced, as evidenced by an increase in specific surface area from 5.9 m^2^ g^−1^ to 80.1 m^2^ g^−1^. However, manganese oxide loading did not exhibit a positive correlation with the specific surface area, as the highest loading led to a reduction in the surface area to 44.5 m^2^ g^−1^. Ying et al.^79^ and Faheem et al.^80^ also demonstrated that the excessive loading of manganese oxides results in a reduction in the specific surface area of biochar. Several studies have also demonstrated that the loading of manganese oxides leads to a reduction in both the specific surface area and average pore size of biochar, with a greater decrease observed at higher loadings; the decrease in specific surface area can range from 204.0 m^2^ g^−1^ to 2.1 m^2^ g^–1.^^81^ During the loading process, KMnO_4_ reacts with the carbon matrix, consuming part of the carbon and producing manganese oxide nanoparticles. At lower contents, the oxides generated are well dispersed, helping to open blocked pores and increase the specific surface area. However, when the content is excessive, continuous carbon consumption and dense oxide deposition lead to partial pore blockage, while excess particles tend to agglomerate, reducing their dispersibility and further blocking the porous network. Therefore, when the loading amount reaches a certain threshold, manganese oxide particles tend to accumulate within the pores of the biochar, filling the original porous structure and thus reducing the specific surface area. These combined effects account for the change in surface area with increasing manganese oxide content. During the manganese oxide loading process, manganese ions (Mn^2+^ or Mn^4+^) react with carbon groups on the biochar surface through oxidation reactions, generating new functional groups, particularly -OH and -COOH, whose contents can increase substantially.^23^ Moreover, these functional groups increase the polarity of biochar, significantly improving its adsorption performance for polar pollutants, such as Sb and Cd. Moreover, the high oxidation state of manganese (Mn^7+^) promotes oxidation reactions on the biochar surface, leading to the production of additional C=O functional groups. The formation of these double bonds not only increases surface polarity but also enhances the resistance of biochar to contamination. Chemical interactions between biochar and pollutants contribute to a more stable and efficient adsorption process. Additionally, manganese oxides can interact with oxides on the biochar surface or with oxygen atoms in functional groups to form stable metal-oxygen bonds (Mn−O−C). These complexes not only alter the functional group structure of biochar but also enhance the specific adsorption of pollutants. For instance, in the case of heavy metal ions, the synergistic effect between manganese oxides and surface functional groups facilitates more effective capture and immobilization, thereby improving the immobilization rate for heavy metals. Furthermore, during the loading process, aromatic functional groups, including benzene rings and carbon−carbon double bonds (C=C), may undergo oxidation. At higher loading levels, oxidation becomes more intense, potentially leading to the destruction of aromatic ring structures. This change reduces the hydrophobicity of biochar, promoting the adsorption and immobilization of polar pollutants.

## Applications of manganese oxide-modified biochar

### Removal mechanism of heavy metals by manganese oxide-modified biochar

**Figure 3:**
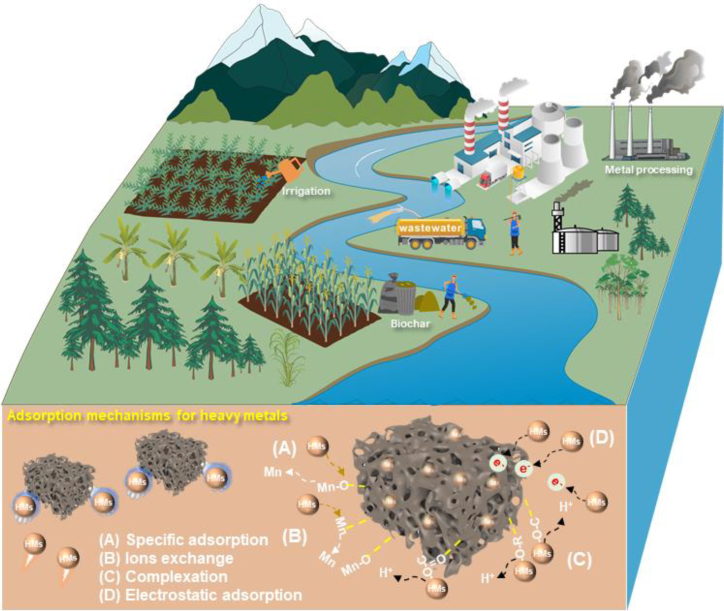
Mechanisms of the adsorption of heavy metals by MBC and their sources from the literature (A) Specific adsorption through the interaction of heavy metals with surface functional groups. (B) Ion exchange between heavy metals and exchangeable ions on the MBC surface. (C) Complexation involving coordination between heavy metals and oxygen-containing groups. (D) Electrostatic adsorption driven by charge interactions between MBC and heavy metal species.

MBC is effective at adsorbing and immobilizing heavy metals due to its ability to form inner-sphere complexes, which enable surface adsorption, ion exchange, and redox reactions (Table 2; Figure 3). Notably, the data presented in Table 2 were obtained under different experimental conditions and are therefore not directly comparable. The functional groups, including -SH, -OH, and -NH_2_, on modified biochar surfaces significantly enhance heavy metal ion adsorption and attract heavy metals via electrostatic or van der Waals forces.^58^^,^^60^ Mn^2+^, Mn^3+^, and Mn^4+^ active sites interact with heavy metal ions, enhancing the adsorption rate. Additionally, manganese ions in MBC are capable of undergoing ion exchange with divalent heavy metals in soil and aqueous environments, thereby facilitating the efficient removal of these contaminants. For example, Liu et al.^82^ utilized ion exchange and metal-oxygen bonding mechanisms between Mn and Cd/Pb to achieve efficient adsorption, with adsorption capacities of 164.6 and 36.8 mg g^−1^ for Cd and Pb, respectively. In addition to interface adsorption and ion exchange, the redox reaction of manganese oxides plays an important role in removing heavy metals by oxidizing or reducing As and Cr to less toxic valence states.^83^ The surface of manganese oxide contains highly reactive oxygen vacancies, which can selectively adsorb heavy metal ions, generate strong chemical interactions, and preferentially capture metals with high electronegativity or specific chemical affinity for Mn.^84^ Spectroscopic analyses, such as XPS and FTIR analyses, have further confirmed the presence of Mn-*O*-metal bonds and the transformation of functional groups, providing direct evidence for inner-sphere complexation and redox mechanisms. Additionally, oxygen atoms or -OH groups on the manganese oxide can directly form covalent or coordination bonds with heavy metals, creating strong inner-sphere complexes without the involvement of water molecules. This direct bonding tightly anchors heavy metal ions to the biochar surface and typically exhibits high selectivity. Such complexation not only enhances the specific adsorption of heavy metals but also improves their adsorption stability, preventing their rerelease into the environment. In multimetal systems, competitive adsorption may occur. For example, Pb is often preferentially adsorbed over Cd due to its stronger affinity for MnO_x_ sites, which should be considered when applying MBC in complex contaminated matrices. In summary, this type of modified biochar significantly increases the surface structure and site activity, primarily through the introduction of manganese oxide. Additionally, it possesses redox capability, and its inner-sphere complexation mechanism results in the production of stable complexes with heavy metals, significantly limiting their bioavailability and mobility.

**Table 2:** Adsorption performance and mechanisms of heavy metals by manganese oxide-modified biochars

Name	Heavy metal	Concentration (mg L^−1^)	Solution pH	Adsorption capacity (mg g^−1^)	Mechanisms	Reference
MPB	As	10	7.0	0.59	Inner-sphere complexation	Wang et al.^47^
	Pb	50	5.5	4.91		
BPB	As	10	7.0	0.20		
	Pb	50	5.5	2.35		
BC@MnO_2_	Pb	5–200	4.5	351.4	Complexation effect of -OH and -COOH groups with heavy metal	Zhang et al.^49^
	Cd			151.4		
	Cu			103.9		
	Zn			68.4		
BCHMn	Pb	250	6.0	108.2	Mineral precipitation, ion exchange, surface complexation, and π electron coordination	Zhou et al.^85^
BCHMn	Cd			312.2		
HMBC	Pb	1000	–	164.6	Ion exchange and metal oxygen bond	Liu et al.^82^
	Cd	200		36.8		
BC-Mn	Pb	100	3.0	214.4	-OH group facilitated the adsorption Pb and Cd, while Mn facilitated As adsorption	Zeng et al.^86^
	Cd	100		165.7		
	As	100		234.9		
Mn-BC	Pb	50	3.0	46.4	The adsorption mechanisms were attributed to high surface area, low zeta potential, and loaded MnO_x_	Wang et al.^87^
	Cd			80.2		
	Ni			18.7		
KM/biochar	Pb	350	5.0	144.5	Precipitation, ion exchange, electrostatic attraction and π-π interaction	Chang and Li^54^
MBR	Pb	150	–	127.8	Specific adsorption provided by Mn-O	Liang et al.^51^
	Cd	50		127.8		
HMO-K-BC	Pb	500	5.0	112.0	Inner-sphere complexation	Wan et al.^88^
	Cd			225.0		
CPMn	Fe	280	–	70.7	Synergistic effects between the -COOH groups and MnO_2_ on the surface of CPMn	Maneechakr et al.^89^
	Ca	30		5.1		
	Zn	75		22.4		
MnB	Cd	100	6.0	23.2	Electrostatic interaction, co-precipitation, π-π interaction, complexation, and ion exchange	Kang et al.^90^
	Zn			10.7		
NMBC	Cu	127.1	6.0	142.0	-OH was consumed and formed mono- or multidentate inner-sphere complexes with Cu	Zhou et al.^91^
BC-MnO_x_	EDTA-Cu	100	8.5	–	EDTA-Cu was attacked by HO· and then adsorbed onto the surface of MnO_x_	Zhu et al.^92^
BC-MnO_x_	CuCA	10	7.8	7.6	CuCA was catalytically oxidized to low molecular weight organic acid and Cu ions were adsorbed by BC-MnO_x_	Zhu et al.^93^
MBC	Ni	150	6.0	105.1	The -NH_2_ and -OH provided react with Ni to form complexation	An et al.^94^
c-PMB/MnO_2_	As	75	3.0	33.5	The components of c-PMB/MnO_2_, including MnO_2_ and biochar, contributed to As and Cr adsorption	Choi et al.^83^
	Cr	52		9.6		
MOLBC	Pb	250	5.0	86.5	The adsorption of Pb was controlled by nano sized manganese oxides, functional groups, π-clouds system, and vacancy defect of biochar graphenic basal plane	Faheem et al.^80^
MnS-fBC	Cd	200	–	–	The removal mechanism of Cd was mixed of adsorption and redox reaction	Fan et al.^95^
OCF-MnO_2_	U	2142	3.0	904.0	MnO_2_ on the surface of biochar enhanced the affinity with U	Ioannou et al.^96^
δ-MnO_2_/BCs	Cu	500	–	230.0	Film and pore diffusions, physisorption, and endothermic mechanisms contributed to Cu removal	Jung et al.^97^

MBP, manganese oxide-modified pine biochar; BPB, birnessite-modified pine biochar; BC@MnO_2_, manganese dioxide nano particles-loaded biochar; BCHMn, nano-MnO_x_ modified biochar; BC-Mn, rice straw loaded with manganese; Mn-BC, biochar modified by KMnO_4_; KM/biochar, KMnO_4_-modified biochar; MBR, MnO_2_-biochar; HMO-K-BC, manganese oxide-based composite; CPMn, KMnO_4_-modified biochar; MnB, KMnO_4_-activated biochar; NMBC, nano MnO_2_-modified biochar; BC-MnO_x_, nanomanganese oxide-modified biochar; MBC, modified peanut shell biochar; c-PMB/MnO_2_, hierarchical corolla-like MnO_2_-decorated porous magnetic biochar composite; MOLBC, MnOx loaded biochar; MnS-fBC, manganese sulfide loaded functional biochar; OCF-MnO_2_, MnO_2_-biochar composites; δ-MnO_2_/BCs, hierarchical birnessite-type MnO_2_/biochar composites.

The processes by which heavy metals are adsorbed by MBC differ between soil and water environments. In aqueous systems, biochar captures heavy metals through specific mechanisms, such as the oxidation of soluble heavy metals by manganese oxide into insoluble forms, leading to precipitation and removal. In soil, rather than being completely removed, heavy metals become less bioavailable, primarily through physical and chemical processes. The addition of MBC results in the formation of stable metal complexes or precipitates with heavy metals, limiting their mobility between soil and plants. Environmental factors, including pH and soil water content, also influence soil-bound heavy metals. For example, a decrease in soil pH following long-term remediation may increase heavy metal mobility. Moreover, aging processes such as MnO_x_ reduction, MBC surface oxidation, and competitive ion displacement may decrease the immobilization efficiency, highlighting the need for long-term monitoring. Therefore, the adsorption and immobilization of heavy metals in soil only reduce bioavailability for a limited period and do not entirely eliminate environmental risks. For effective management, water quality should be regularly tested after remediation to ensure that MBC does not release excessive manganese into water bodies. Similarly, soil heavy metal concentrations should be monitored over time to assess mobility, environmental risks, and the long-term efficacy of remediation efforts.

### Removal mechanism of organic pollutants using manganese oxide-modified biochar

**Figure 4:**
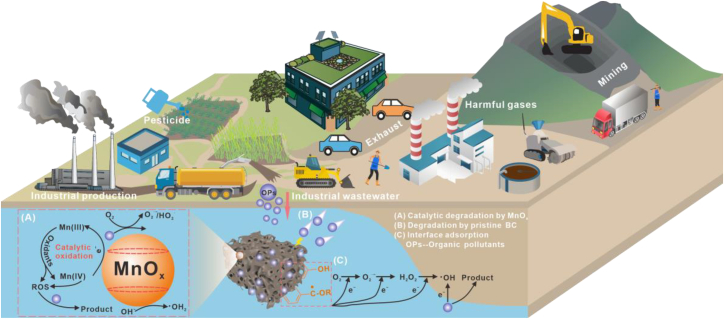
Mechanisms of the adsorption of organic pollutants by MBC and their sources from the literature (A) Generation of reactive oxygen species (ROS) by manganese oxides, leading to the oxidative degradation of organic pollutants. (B) Electrostatic adsorption between charged organic pollutants and the MBC surface. (C) Pollutant degradation mediated by hydroxyl radicals produced during the reaction process.

Manganese oxides are abundant natural oxidants in soils and are typically present at concentrations of 500–1000 mg kg^−1^; however, their forms and concentrations vary depending on soil type, geological context, and chemical conditions.^25^^,^^98^^,^^99^ The manganese oxide content is low in acidic soils due to the reduction of Mn^4+^ to Mn^2+^ under acidic conditions. Additionally, protons from organic acids, such as humic, citric, and oxalic acids, interact with oxygen on the manganese oxide surface, weakening the metal‒oxide bond and promoting oxide dissolution, which accelerates reduction. In alkaline or neutral environments, manganese oxides are abundant, particularly under oxidative conditions where Mn^2+^ is oxidized to Mn^3+^ or Mn^4+^ (Table 3). High-valent manganese can engage in electron transfer with organic matter, reducing manganese oxides to lower valence states while simultaneously oxidizing organic compounds.^100^ This process alters the molecular structure of organic matter, leading to the formation of simpler, biodegradable molecules or the mineralization of organic matter into H_2_O and CO_2_. The pore structure of biochar and the large surface area of manganese oxides provide abundant active sites, which effectively adsorb organic matter and facilitate surface oxidation reactions, thereby enhancing organic matter decomposition (Figure 4).^101^^,^^102^

**Table 3:** Adsorption performance and mechanisms of organic pollutants by manganese oxide-modified biochars

Name	Organic pollutant	Oxidant	Reaction conditions	Removal efficiency	Mechanisms	Reference
Mn_x_O_y_@BC	Oxytetracycline	Periodate	PI = 0.25 mmol L^−1^; OTC = 20 mg L^−1^; T = 25 °C	97.5%	Periodate was activated by Mn_x_O_y_@BC and the generated O_2_·^–^, 1O_2_, and ·OH degraded OTC	Fang et al.^103^
KMnO_4_/biochar	4-nitrophenol	KMnO_4_	KMnO_4_ = 150 μmol L^−1^, 4-nitrophenol = 10 μmol L^−1^, T = 25 °C	92.0%	Oxidation of 4-nitrophenol by Mn^6+^/Mn^5+^ oxidants produced by KMnO_4_/biochar	Tian et al.^104^
Mn-BC	Tetracycline	–	TC = 200 mg L^−1^, T = 25 °C	∼80.0%	Manganese oxide acted as an oxidizer on accelerating the removal of TC	Shen et al.^105^
MnO_x_/biochar	Atrazine	O_3_	O_3_ = 20 mg L^−1^, ATZ = 10 μmol L^−1^, T = 20 °C	83.0%	The loaded manganese oxide increased *Lewis* acid sites and induced O_3_ decomposition to generate ·OH	Tian et al.^106^
MnO_2_@BRB	Phenol	KMnO_4_	KMnO_4_ = 100 μmol L^−1^, Phenol = 10 μmol L^−1^, T = room temperature	73.4%	*In-situ* generated manganese oxide was complexed with biochar to catalyze phenol removal by KMnO_4_	Li et al.^107^
MBC	Tetracycline	–	500 mg L^−1^ of antibiotics	35.6%	The π-π interactions and surface coordination adsorbed antibiotics pollutants produced from MBC	Liao et al.^108^
	Norfloxacin			4.5%		
	Sulfamethoxazole			1.9%		
Mn-BBC	Orange II	Peroxymonosulfate	PMS = 2 mmol L^−1^, AO7 = 20 mg L^−1^, T = 25 °C	35.1%	The produced Mn^3+^ and PMS derived the Orange II degradation by electron-transfer processes	Gao et al.^109^
Mn-BC	Oxytetracycline	–	OTC = 0.22 mmol L^−1^, T = room temperature	>90.0%	The produced persistent free radicals through manganese-doped degraded OTC	Dai et al.^57^
Mn@BC	Methylene blue	NaIO_4_	PI = 1 mmol L^−1^, MB = 60 mg L^−1^, T = 25 °C	100%	The free radicals and non-free radicals generated by the reaction of MnO_x_ with PI degraded MB	Gong et al.^110^
ASMn-Nb	Orange II	Na_2_S_2_O_8_	PMS = 1.6 mmol L^−1^, AO7 = 20 mg L^−1^, T = 25 °C	100%	Non-radicals, radicals, and MnO_x_ acted as reactive sites for AO7 degradation	Mian et al.^111^
MnO_2_@SBC	Methylene blue	Peroxymonosulfate	PMS = 0.5 mmol L^−1^, MB = 35 mg L^−1^, T = 25 °C	>85.0%	SO_4_^−^·, ·OH, and ^1^O_2_ were contributed for the degradation MB	Li et al.^112^
nMnO@BcB	COD	H_2_O_2_	–	94.5%	·OH produced by Fenton oxidation process degraded COD	Singh et al.^113^

Mn_x_O_y_@BC, manganese oxide/biochar composites; KMnO_4_/biochar, KMnO_4_ modified biochar; Mn-BC, manganese dioxide modified biochar; MnO_x_/biochar, MnO_x_-loaded biochar; MnO_2_@BRB, MnO_2_ complexed with biochar; MBC, manganese oxide-loaded biochar; Mn-BBC, Mn-incorporated bacterial-derived biochar; Mn-BC, Mn-modified biochar; Mn@BC, manganese oxide/biochar composites; ASMn-Nb, sludge derived carbon-supported MnO_x_; MnO_2_@SBC, MnO_2_ loaded biochar; nMnO@BcB, nMnO modified biochar.

In contrast to heavy metals, organic pollutants encompass a wide range of compounds, primarily with macromolecular structures. Each type of organic pollutant exhibits distinct characteristics, making effective removal challenging when conventional adsorbents are used. Antibiotic pollutants are categorized into penicillin, macrolides, fluoroquinolones, and tetracyclines and primarily derive from the overuse of antibiotics in healthcare, aquaculture, and wastewater treatment.^101^^,^^114^ Some antibiotics also have the ability to spread resistance genes, which can easily accumulate in water, soil and sediments, causing environmental damage. Gao et al.^115^ synthesized nano-manganese oxide-modified biochar to evaluate its efficiency in adsorbing and removing n-butyl phthalate and oxytetracycline and reported that higher loading amounts increase the content of surface functional groups and that electron transfer from Mn^4+^ to Mn^3+^ and Mn^2+^ drives pollutant degradation. Shen et al.^105^ demonstrated that MBC synthesized via coprecipitation is an effective adsorbent for tetracycline removal and revealed that the degradation pathway involves hydroxylation, hydrolysis, and N-methyl decomposition. Other organic pollutants include organic dyes, such as azo dyes, anthraquinone dyes, and acid/basic dyes, most of which exhibit poor biodegradability, particularly those containing N=N double bonds or anthraquinone structures, which are highly stable. Iqbal et al.^116^ used nano-zero-valent manganese-modified biochar to degrade Congo red with 77.0% efficiency; moreover, the system activated H_2_O_2_ to generate ·OH, achieving over 95% pollutant removal. In addition, we have further emphasized that different manganese oxide crystal structures, such as δ-MnO_2_ and birnessite, exhibit distinct reactivities toward organic pollutants. The presence of Mn(III) intermediates and reactive oxygen species (ROS), including ·OH, ·O_2_^−^, and ^1^O_2_ plays a crucial role in the oxidative degradation process, with MnO_x_ simultaneously functioning as both an oxidant and as an electron shuttle to accelerate electron transfer. These radical-mediated pathways contribute to the cleavage of -H, C=C, and aromatic bonds in complex organic molecules, thereby increasing the mineralization efficiency. Therefore, relying solely on manganese oxides for organic matter degradation rarely produces ideal results in the environment, although synergistic effects with other oxides can further enhance degradation. Periodate, persulfate, H_2_O_2_, and KMnO_4_ are common oxidants that generate free radicals or reactive oxygen species under various conditions, thereby significantly contributing to the breakdown of organic pollutants. For example, periodate generates active species such as IO_3_^−^ or I· under acidic conditions, which efficiently break C-H, C=C, and other chemical bonds in organic pollutants, triggering chain reactions. Fang et al.^103^ utilized Mn_x_O_y_@BC to activate periodate for oxytetracycline (OTC) removal from water, achieving a 97.5% OTC removal rate, and demonstrated that ·O_2_^−^, ^1^O_2_, and ·OH are the primary species responsible for organic matter degradation. Jung et al.^117^ employed MBC as a catalyst to degrade bisphenol A via a heterogeneous Fenton process. The combination of manganese oxide and ultrasonication induced free radical generation and increased the interfacial mass transport efficiency between the solid and liquid phases. In contrast, KMnO_4_ has strong oxidative properties under acidic conditions and undergoes redox reactions with organic pollutants. MnO_4_^−^ facilitates the electron transfer-mediated oxidation and consequent degradation of organic matter. The integration of biochar with catalytic oxidants significantly enhances the degradation and removal of organic matter. Nevertheless, challenges remain in the practical application of MBC. The dissolution of Mn^2+^ during prolonged operation may lead to secondary contamination, and the incomplete oxidation of organic matter can generate toxic intermediates. Therefore, continuous monitoring of manganese release and byproducts is essential to ensure environmental safety. To address these limitations, synergistic applications integrating MBC with light irradiation, ultrasound, or electrochemical systems have been proposed, which substantially boost radical generation and increase degradation efficiency.

## Biochar regeneration and treatment after use

### Common regeneration technique

Biochar is widely used in soil amendment, water purification, and pollution control, and its regeneration and recycling hold significant environmental importance. Currently, the primary methods for biochar regeneration include thermal treatment, acid and alkali leaching, chelating agent leaching, and microbial degradation.^118^^,^^119^ Heat treatment, a physical regeneration method, involves heating biochar at 300°C–600°C to remove organic matter adsorbed on the surface, which effectively restores its pore structure, increases its specific surface area, and mitigates the decline in adsorption performance. Qiao et al.^120^ demonstrated that the rate of biochar regeneration consistently increased as the temperature increased from 80 °C, whereas the initial adsorption capacity remained at or below 48%, primarily due to the enhanced surface site activity induced by a series of reactions occurring at elevated temperatures. However, high temperatures can alter the form of the loaded manganese oxides, leading to changes in the final products. Therefore, controlling the heat treatment temperature is essential to minimize both costs and structural damage to the biochar. Inorganic acids and bases, such as HCl, H_2_SO_4_, and NaOH, are commonly employed as desorbents in biochar regeneration.^121^^,^^122^^,^^123^^,^^124^^,^^125^ Acidic desorbents increase the H^+^ concentration on the biochar surface through protonation, facilitating the desorption of heavy metals via competitive reactions. Furthermore, under acidic conditions, neutralization of the negative surface charge of biochar reduces the electrostatic attraction between biochar and adsorbates, which promotes pollutant desorption.^126^ Alkaline desorbents supply OH^−^ ions, which react with cations in biochar to form soluble complexes.^127^ Under alkaline conditions, the dissociation of functional groups is enhanced, increasing the negative charge on the biochar surface, which in turn increases cation repulsion and facilitates the desorption of adsorbed substances. Regenerating biochar with inorganic acids or bases as desorbents reduces environmental costs and enhances the recovery of adsorption capacity. Active groups are introduced, improving the effectiveness of environmental remediation. However, acid treatment can lower the pH, compromising stability during heavy metal adsorption and immobilization, and changes in environmental pH can facilitate the rerelease of adsorbed heavy metals. In contrast to acids and bases, chelating agents (e.g., EDTA, CA, and HEDTA) are widely employed for heavy metal desorption due to their strong complexation ability. These agents contain functional groups (-OH, -COOH, or -NH_2_) that bind metal ions through coordination bonds, forming complexes that are more soluble in water than the original metal ions.^128^^,^^129^^,^^130^ The desorption efficiency of chelating agents is influenced by the solution pH. In acidic environments, heavy metals are more easily desorbed in their free form, and the addition of chelating agents introduces competing ions that compete for binding sites, further promoting desorption. While chelating agents offer high efficiency and versatility in heavy metal desorption, they also present environmental concerns, such as toxicity and persistence, as well as high economic costs. Future efforts should focus on the research and development of new green desorbents, such as NaCl and CaCl_2_, as well as the combined use of microorganisms and intelligent control systems^131^^,^^132^; these approaches will enhance the efficiency and sustainability of heavy metal pollution remediation and offer more effective solutions for environmental protection.

### Environmental effect and treatment of waste biochar

Biochar can significantly enhance environmental quality, but its widespread use warrants the careful consideration of its environmental impacts, which can be both beneficial and detrimental.^133^^,^^134^^,^^135^^,^^136^^,^^137^^,^^138^ The scientific application of biochar relies on an understanding of the underlying mechanisms to reduce potential risks.^139^^,^^140^ Biochar is aromatic and has a stable structure, with properties that are influenced by the biomass type, and it generally improves soil physicochemical properties and structures.^141^^,^^142^^,^^143^^,^^144^ For example, the porous structure of biochar enhances soil porosity, improves aeration, and facilitates the supply of oxygen to roots, particularly in compacted or poorly drained soils.^145^^,^^146^^,^^147^ Additionally, biochar improves soil fertility and plant growth by increasing the cation exchange capacity through its negatively charged surface.^148^^,^^149^^,^^150^ Biomass materials, such as rice husks, crab shells, and banana straws, are rich in mineral elements, which provide nutrients to plants as they are absorbed by the soil.^151^ However, in some cases, the application of biochar can alter soil pH.^152^^,^^153^^,^^154^ Specifically, in acidic soils, biochar can significantly increase the pH, which may have a considerable effect on plant growth. The application of biochar can also result in soil salt accumulation, particularly in environments with poor water management, where an excess of sodium ions can lead to soil salinization, thereby affecting soil structure and plant water absorption. Additionally, biochars derived from sludge and iron slag may contain highly active heavy metals, which can leach out during application and exacerbate heavy metal contamination.

Although studies suggest that the addition of biochar can create a more favourable environment for soil microorganisms and increase their diversity and abundance, the overall impact on soil microbial communities remains intricate and multifaceted.^146^^,^^155^^,^^156^^,^^157^ Generally, biochar possesses micro- and mesoporous structures, providing abundant attachment sites for soil microorganisms and thereby promoting the aggregation and proliferation of microbial communities.^158^ Some research has shown that the utilization of biochar in soil significantly increases the abundance of beneficial microorganisms, such as nitrogen-remediating bacteria, phosphorus-solubilizing bacteria, and organic matter-decomposing bacteria.^159^^,^^160^^,^^161^^,^^162^ This, in turn, enhances soil fertility, promotes plant growth, and improves the ecological functions of the soil. Moreover, biochar can modify soil redox potential and pH levels, thereby indirectly affecting the structure of microbial communities. However, biochar application may also suppress the growth of certain soil pathogens, particularly when excessive organic carbon in the soil is decomposed by microorganisms, potentially enabling excess growth of harmful bacteria. Additionally, heavy metals and undecomposed organic matter in biochar can be toxic to soil microorganisms. In cases of soil pollution, excessive biochar application may lead to the accumulation of heavy metals, which in turn impairs the growth and activity of microorganisms.^17^^,^^77^

To mitigate potential negative effects, several measures must be implemented to optimize biochar use. First, the application rate must be carefully controlled to prevent issues such as soil salinization, an excessive increase in pH, or toxic substance accumulation. A thorough physical and chemical analysis of the soil should be conducted prior to application to guide the development of a tailored application plan. The source and preparation method of biochar are also critical factors influencing its quality. Using high-quality, biodegradable biochar is crucial for minimizing adverse effects. For instance, selecting low-pollution, mineral-rich feedstocks and employing low-temperature pyrolysis can reduce the contents of toxic substances, thus lowering the potential toxicity to soil and microorganisms. Additionally, biochar application should be combined with other agricultural practices, such as organic fertilization and soil pH regulation, to promote soil health and support plant growth, ultimately enhancing soil function and ecological service capacity. Long-term monitoring is essential for tracking changes in soil properties and microbial communities to allow for timely adjustments to application strategies. These measures offer a rational approach to agricultural waste utilization and pollution remediation, benefiting both environmental sustainability and resource efficiency.

## Future research trends and challenges

Unlike other modified biochars, MBC has exceptional adsorption effects on both cationic and anionic heavy metals. Its primary mechanism involves heavy metal immobilization through inner-sphere complexation rather than ion exchange, and MBC demonstrates strong adsorption-recycling performance. Additionally, under specific conditions, MBC can generate free radicals that facilitate the degradation of organic matter in the environment, further promoting its practical application. MBC has been increasingly applied in the remediation of soil and water pollutants and exhibits excellent performance and stability. However, numerous challenges still require further investigation in future studies.

1 The long-term stability of manganese oxides in the environment is a critical factor influencing their effectiveness as remediation materials. Our team carried out an actual remediation project in 2023 for heavy metal-contaminated soil, with an area of approximately 1600 m^2^. After 7 days of treatment with MBC, the efficiencies of Cd and Zn remediation reached 86.1% and 83.4%, respectively. Follow-up sampling revealed that the available fractions of Cd and Zn in the soil decreased by 82.8% and 84.1%, respectively, indicating that MBC maintained excellent stability under conventional conditions. In addition, the application of MBC only caused a slight increase in soil pH, with an observed increase of approximately 0.5 units under high loading. Although numerous studies have indicated the high remediation performance of MBC in the short term, its redox properties can be affected by environmental factors such as pH, temperature, and humidity. Manganese oxides in soil environments can be reduced or dissolved, resulting in the loss of surface activity and remediation effects. Furthermore, Mn dissolution may result in high Mn concentrations in soil or water, adversely affecting the ecosystem and water quality. Therefore, future research should address the long-term stability of manganese oxides, with a particular focus on their stability in complex environments. Moreover, it is essential to assess the long-term effects of MBC on soil and aquatic ecosystems to ensure that the use of MBC for remediation does not introduce new environmental pollution.
2 The remediation mechanisms of MBC are responsible for its effectiveness but are incompletely understood. Previous studies have demonstrated that manganese oxides can remove heavy metals or organic pollutants through adsorption, reduction, and oxidation, but the remediation mechanisms corresponding to various pollutants and environmental conditions remain unclear. In particular, the effects of the surface structure of biochar and the bonding modes of manganese oxides on the remediation capacity still require further exploration. The manganese oxide types (e.g., α-MnO_2_, β-MnO_2_, and δ-MnO_2_) influence the removal efficiency for pollutants; therefore, the effects of different manganese oxide types and their surface properties deserve further investigation. In addition, environmental factors such as ionic strength and temperature can affect the remediation potential for manganese oxides, and these factors must be carefully considered in experimental design to ensure practical effectiveness.
3 Although MBC has demonstrated excellent remediation effects in laboratory studies, realizing its large-scale application in natural environments remains challenging. First, the MBC preparation process can carry high costs, particularly in the selection and treatment of manganese sources. Reducing costs and improving production efficiency for industrial-scale applications presents a critical challenge. Second, more research should focus on improving the application benefits and cost-effectiveness of MBC. Finally, in large-scale applications, the effectiveness and stability of manganese oxides must be maintained to effectively remediate the target pollutants and prevent secondary pollution.
4 Currently, MBC is primarily used to remediate single pollutants, such as heavy metals or organic contaminants, but natural environments typically exhibit complex pollution characteristics. Composite pollution can affect the performance of remediation materials in complex ways, particularly through interactions between pollutants that influence their adsorption properties, redox properties, and behavior in the context of other remediation mechanisms. Therefore, the remediation efficacy of MBC for complex pollution, such as systems with coexisting heavy metals and organic pollutants, requires further investigation. Extensive experiments and mechanistic studies are required to assess the potential and limitations of MBC in complex-pollution environments, particularly with respect to competitive adsorption and the combined removal of various pollutants.
5 The manganese oxide loading amount and the surface properties of biochar significantly affect remediation performance. It is critical to optimize the manganese oxide loading to achieve the best remediation effect while preventing excessive manganese oxides from causing secondary pollution or instability in the soil. In addition, the surface properties of biochar, including pore structure, mineral components, and surface functional groups, markedly influence the loading of and remediation capacity for manganese oxides. Future research should focus on optimizing the loading mode and amount of manganese oxides by modifying the structures and functional groups of biochar to increase the remediation efficiency.
6 Manganese oxides possess strong oxidizing ability, enabling the degradation of organic pollutants or other pollutants in redox reactions by releasing oxidative free radicals such as ·OH and O_2_^−^. However, the catalytic activity of manganese oxides is affected by their crystal form, surface conditions, and electronic structure. Different types of manganese oxides, including δ-MnO_2_, γ-MnO_2_, and α-MnO_2_, present quite different activities in catalytic oxidation reactions. Optimizing the catalytic activity of manganese oxides by regulating their structure and surface properties is a key research direction. Moreover, the oxidation mechanism of manganese oxides remains incompletely understood, and further exploration is needed to elucidate the mechanisms involved in catalytic oxidation reactions, including studies of the generation of oxidants, identification of reaction intermediates, and detailed analysis of the reaction pathway.
7 Currently, there are no standardized guidelines for the selection of raw materials, preparation methods, or related parameters for MBC. The technical approaches proposed in various studies remain unstandardized, and standardized criteria are needed for evaluating the performance and cost effectiveness of modified biochar.v

## Conclusions

This review provides a detailed introduction to the crystal structure and adsorption activity of manganese oxide and the potential applications of manganese oxide-modified biochar in environmental contexts. It summarizes the adsorption behaviors and mechanisms of pollutants under various environmental conditions, offering valuable insights to support research and development related to agricultural waste utilization and pollution remediation techniques, ultimately contributing to environmental sustainability. The reaction between KMnO_4_ and carbon in biomass presents significant environmental benefits. This process not only produces weakly crystalline manganese oxide with high adsorption activity but also converts carbon into carbonate while introducing numerous functional groups capable of complexing with heavy metals.

## Data and code availability

Data will be made available on request.

## Acknowledgments

This work was supported by the Key R&D Program of Guangxi (Grant No. AB22035038) and 10.13039/501100012434Middle-aged and Young Teachers' Basic Ability Promotion Project of Guangxi (Grant No. 2025KY0106). We would like to express our gratitude to the Key Laboratory of Ecology of Rare and Endangered Species and Environmental Protection (Guangxi Normal University), Ministry of Education, for their assistance with the experimental tests. We also wish to thank the Key Laboratory of Medicinal Resource Chemistry and Pharmaceutical Molecular Engineering (Guangxi Normal University) for providing the testing instruments.

## Author contributions

Anyu Li: writing-original draft, data curation, investigation, software, and methodology. Song Lei, Shujia Chen, Xiaotong Zhou, Zhihua Wu, and Feng Jiang: writing-review and editing and investigation. Peng Zheng: methodology, writing-review and editing. Lening Hu: methodology and investigation. Hua Deng: conceptualization, funding acquisition, supervision, and writing-review and editing.

## Declaration of interests

The authors declare that they have no known competing financial interests or personal relationships that could have appeared to influence the work reported in this article.
